# The Association of Weight Status with Physical Fitness among Chinese Children

**DOI:** 10.1155/2010/515414

**Published:** 2010-06-27

**Authors:** Xianwen Shang, Ailing Liu, Yanping Li, Xiaoqi Hu, Lin Du, Jun Ma, Guifa Xu, Ying Li, Hongwei Guo, Guansheng Ma

**Affiliations:** ^1^National Institute for Nutrition and Food Safety, Chinese Center for Disease Control and Prevention, 29 Nan Wei Road, Beijing 100050, China; ^2^School of Public Health, Peking Union Medical College, 9 Dong Dan 3 Tiao, Beijing 100730, China; ^3^Guangzhou Center for Disease Control and Prevention, 23 Zhong Shan San Lu, Guangzhou 510080, China; ^4^Beijing University Health Science Center, 38 Xue Yuan Road, Beijing 100191, China; ^5^Shandong University, 44 Wen Hua Xi Lu, Jinan 250012, China; ^6^Public Health College, Haerbin Medical University, 157 Bao Jian Road, Haerbin 150081, China; ^7^Fudan University, 138 Yi Xue Yuan Lu, Shanghai 200032, China

## Abstract

*Objective*. To investigate the association of weight status with physical fitness among Chinese children. *Methods*. A total of 6929 children aged 6–12 years were selected from 15 primary schools of 5 provincial capital cities in eastern China. The height and fasting body weight were measured. The age-, sex-specific BMI WHO criteria was used to define underweight, overweight and obesity. Physical fitness parameters including standing broad jump, 50 m sprint, and 50 m^∗^8 shuttle run were tested. *Results*. The prevalence of underweight, overweight, and obesity was 3.1%, 14.9%, and 7.8%, respectively. Boys performed better than girls, and the older children performed better than their younger counterparts for all physical fitness tests. No significant difference in all three physical fitness tests were found between children with underweight and with normal weight, and they both performed better than their counterparts with overweight and obese in all three physical fitness tests. The likelihood of achieving good performance was much lower among overweight and obese children in comparison with their counterparts with normal weight (OR = 0.13–0.54). *Conclusions*. An inverse association of obesity with cardiorespiratory fitness, muscle explosive strength, and speed was identified among Chinese children.

## 1. Introduction

The increasing prevalence of obesity is a major public health problem in both the developed and the developing world [1–3] In Asia, there is an alarming increase in the proportion of overweight and obese children and adolescents especially in countries undergoing nutritional and lifestyle transition, such as China [1, 4]. In 1982, the prevalence of overweight and obese youngsters in China was 1.2% and 0.2%, respectively. The rates increased in triple or more with 4.4% for overweight and 0.9% for obesity in 2002 [1]. 

Childhood obesity is a risk factor for a number of chronic diseases including heart disease, some cancers, and osteoarthritis in adulthood life. Some diseases, however, can become manifest during childhood, particularly type 2 diabetes [5]. In addition, some studies reported that overweight and obesity decreased the physical exercise capability and then reduced health-related physical fitness, such as cardiorespiratory fitness and speed of movement [6, 7]. Maintaining an appropriate level of health-related physical fitness allows a person to participate and enjoy physical activity, and reduce the risk of disease and injury. Report on the Physical Fitness and Health Surveillance of Chinese School Students in 2005, revealed that muscular explosive strength, cardiorespiratory fitness, and speed of movement in Chinese children has been decreasing during the past two decades [8]. With the rapid increase in obesity and decrease in physical fitness among Chinese children, we assume a relationship between overweight/obesity and health-related physical fitness in Chinese children. Some previous studies indicated the relationship between obesity and physical fitness performance in Caucasian children [9, 10]. However, ethnic differences in body composition are evident with a higher %BF, less FFM in Asians than Caucasians at the same BMI [11–14]. Limited study on this relationship was conducted in a large sample in Chinese children. 

Moreover, underweight is still a public health problem in China which is undergoing nutritional and lifestyle transition. It is meaningful to explore the relationship between underweight and physical fitness performance, in addition to obesity. Few data are available in a large sample of Chinese children. Therefore, the purpose of the current paper is to explore the association of underweight, overweight, and obesity with physical fitness among Chinese children.

## 2. Subjects and Methods

Five provincial capital cities in eastern China, including Haerbin, Beijing, Shandong, Shanghai, and Guangzhou were selected for this study. Six primary schools were randomly selected from each selected city. Two classes from each grade from each selected school were randomly selected. All students in the selected classes were recruited as the study subjects. 

This study was approved by the Ethical Review Committee of the National Institute for Nutrition and Food Safety and Chinese Center for Disease Control and Prevention. A written consent from parent and the oral consent from each subject were obtained. 

### 2.1. Anthropometric Measurement

Height was measured to the nearest 0.1 cm in bare feet. Fasting body weight was measured to the nearest 0.1 kg using a balance-beam scale (RGT-140, Weighing Apparatus Co. Ltd. Changzhou Wujin, China) with participants wearing lightweight clothing. All the measurements were taken by trained investigators following standard operation procedure. 

BMI was calculated by dividing weight by the square of height (BMI = weight (kg)/height (m)^2^). 

Underweight, overweight, and obesity was classified according to the WHO age- and sex-specific BMI cut-off points [15].

### 2.2. Physical Fitness Measurements

Three physical fitness tests were measured in our study, including standing broad jump, 50 m sprint, and 50 m*8 shuttle run. The standing broad jump was used to evaluate lower limb explosive strength. Participants stood with the feet immediately behind the starting line and separate from each other approximately with the shoulder's width over a nonslippery and hard surface. Participants jumped as longest as possible with two feet together. The longest jumping distance of triplicate attempts was recorded in centimeters. The 50 m sprint was measured to evaluate the speed of movement. Participants were instructed to run in a straight line and at the highest speed possible. The test was performed once and recorded to the nearest 0.1 s (CASIO, HS-70W stopwatch). The 50 m*8 shuttle run was measured to evaluate the cardiorespiratory fitness and agility. This test required participants to run back and forth 8 times along a track between two poles set 50 m apart at the highest speed possible and to turn round the poles counterclockwise. The test performed once and recorded to the nearest 0.1 s (CASIO, HS-70W stopwatch).

The physical education teachers showed the children how to do the tests in details. In order to encourage all participants to try their best in the physical fitness tests, they were informed that the test results would be recorded as the performance physical education for the semester. All the measures were taken by trained physical education teachers.

### 2.3. Statistical Analysis

Chi-square test was used to compare the age and sex difference in the prevalence of underweight, overweight, and obesity. Continuous variables were described as mean ± standard deviation (SD). *T*-test and one-way analysis of variance (ANOVA) were used to compare the age and gender differences in physical fitness test results. Weight status differences in physical fitness test results were compared using analysis of covariance (ANCOVA) with Bonferroni multiple comparison after controlling age and sex. Odds ratio (OR) was calculated by Cochran-Mantel-Haenszel Statistics to explore the likelihood of good performance (more than age- and gender-specific 75th percentile and 90th percentiles of each physical fitness test result, respectively [16]) in physical fitness tests in underweight, overweight and obese children compared with normal weight children adjusted for age and gender. It was considered significant if *P* value <.05.

## 3. Results

A total of 6929 elementary children (3604 boys, 3325 girls) aged 6–11 years (9.2 ± 1.4 years) were enrolled into the study and completed the anthropometric measurements. A total of 6767 children completed the test of standing broad jump and 6649 children completed 50 m sprint, while 4771 children completed 50 m*8 shuttle run. As all participants in Beijing did not perform the test due to the restriction caused by the epidemic of swine flu during the data collection, high dropout rate in 50 m*8 shuttle run was obtained. No significant differences in age, sex, height, and weight were found between the dropout group and the study sample.

The overall prevalence of underweight, overweight, and obesity was 3.1%, 14.9% and 7.8%, respectively (Table 1). The proportions of overweight and obesity among boys were significantly higher than that among their female counterparts (16.2% versus 13.6%; 10.3% versus 5.1%, respectively). The prevalence of underweight among boys was lower than girls (2.0% versus 4.7%). No significant differences in prevalence of underweight, overweight, and obesity were found among age groups.

The physical fitness test results by age and gender were shown in Table 2. The distance of standing broad jump in boys was significantly longer than that in girls (146 cm versus 137 cm). Boys run significantly faster than girls in both 50 m sprint (10.3 s versus 10.6 s) and 50 m*8 shuttle run (127.2 s versus 130.0 s) across all age groups. Older children performed better in all three physical fitness tests than their younger counterparts within the same gender subgroup.

**Figure 1:**
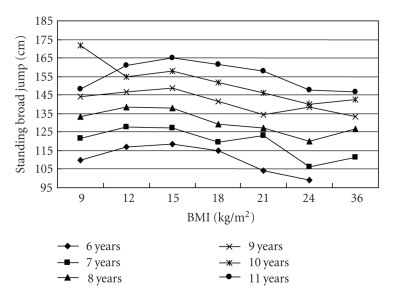
The association of standing broad jump length (cm) with BMI (kg/m^2^) in 6775 Chinese children aged 6–11 years.

**Figure 2:**
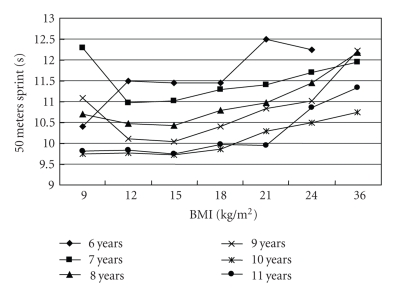
The association of 50 m sprint time (s) with BMI (kg/m^2^) in 6775 Chinese children aged 6–11 years.

**Figure 3:**
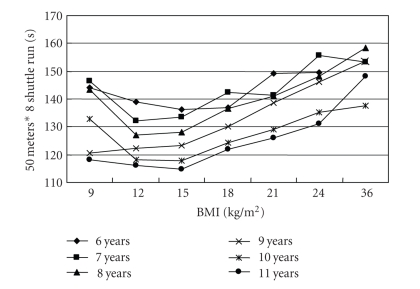
The association of 50 m*8 shuttle run time (s) with BMI (kg/m^2^) in 4771 Chinese children aged 6–11 years.

Table 3 shows the comparisons in physical fitness test results among children with underweight, normal weight, overweight and obesity. No significant differences in all three physical fitness test results were found among different weight status group after controlling for age and gender. No significant differences in all three physical fitness test results between underweight and normal-weight children were found. Both underweight and normal-weight children had higher value in distance of standing broad jump while lower time of 50 m sprint and 50 m*8 shuttle run than their overweight and obesity counterparts. The distance of standing broad jump increased along with the increase of BMI value till to the overweight cut-off points and then decreased along with BMI decrease (*P* < .001 for trend test) (Figure 1). The time of 50 m sprint and 50 m*8 shuttle run decreased along with the increase of BMI till to the overweight cut-offs and then increased with BMI (*P* < .001 for trend test) (Figures 2 and 3).

Table 4 indicates the proportion of children with physical fitness tests results above the age- and gender-specific 75th percentile and 90th percentile by weight status. Less than 9% obese children had a result above the 75th percentile of each physical fitness test, and less than 4% obese children had a result above the 90th percentile of each physical fitness test after adjusted for age and gender. The likelihood of failure to pass the physical fitness tests among overweight children was 2-3 times than their normal weight counterparts. The obese children had about 4, 7, and 8 time risk for no passing (less than 90th percentile) the standing broad jump, 50 m sprint, and 50 m*8 shuttle run, respectively, compared with normal-weight children. No significant increase risk for no passing these tests in underweight children was found compared with normal-weight children.

## 4. Discussion

Our results revealed that the overweight and obese children performed worse in standing broad jump, 50 m sprint and 50 m*8 shuttle run compared with normal weight children. The results are agreement with previous studies. With the accumulation of body fat, explosive strength, cardiorespiratory fitness, speed, and agility of children declines continuously [16–21]. 

Maintaining an appropriate level of health-related physical fitness allows a person to participate and enjoy physical activity and reduce the risk of disease and injury [22, 23]. Health-related physical fitness includes the characteristics of functional capacity, such as muscular strength,cardiovascular endurance and motor ability [24]. In China, similar to other countries, the Physical Fitness and Health Surveillance of Chinese School Students includes the three components of fitness, such as standing broad jump, 50 m sprint, and 50 m*8 shuttle run. Therefore, we selected the three tests to evaluate the health-related fitness in the current study. All of the three tests required propulsion or lifting of body which was disadvantage in overweight and obese children due to the extra body load to be moved while performing these tests. However, overweight and obesity children can perform equally well or even better than children with normal weight in those muscular fitness tests where their body does not have to be transported, such as handgrip strength test [25]. Some studies also showed that obese children had similar cardiovascular fitness to normal-weight children after adjustment for body composition [26]. However, the obese children are inconvenient in mobility and less self-confidence, which makes them to participate in less physical activities and subsequently, the low physical activity level will increase risk for chronic disease.

No significant differences in physical fitness performance between underweight and normal-weight children were found in our study. However, some previous studies indicated underweight children and adolescents had poorer performance for sit-up and sit-and-reach [20], running endurance [27] and push-up [16] than their normal-weight counterparts. One of the main reasons for this inconsistency might be the low grade of the underweight. The difference in mean BMI of underweight and normal weight groups was only about 2 kg/m^2^. Only urban children were involved into our five study sites which are top developed area in China and the prevalence of underweight was low (3.1%).

Boys showed better performance than girls in all fitness tests at all ages, which was similar to the previous studies [28–30]. For example, Pangrazi and Corbin indicated that boys performed better than girls in explosive strength, endurance of muscles, and speed [31]. Consistent with previous studies, the present study also found older children performed better than their younger counterparts [32]. The age and gender differences in physical fitness performance can be explained, in part, by the age and gender difference in body composition. Boys have greater muscle mass, bone density, and less body fat than girl across age groups and older children have greater bone density and muscle mass than younger children [17, 33–35]. Moreover, compared with girls, boys were more physical active [36].

In addition, in the present study, we found the prevalence of overweight and obesity was 14.9% and 7.8%, respectively, in Chinese urban children in 2008, which is much higher than that in 2002 China National Nutrition and Health Survey (CNNHS). In 2002, the prevalence of overweight and obesity in Chinese urban children were 8.5% and 4.4%, respectively [37]. Despite the current sample was less representative than the 2002 CNNHS, the rapid increasing of overweight and obesity can still be evident. 

There are some limitations of the current study. Firstly, only three physical fitness tests were measured which were not able to assess the overall physical fitness. Secondly, Weight status was classified on the basis of BMI in our study. However, BMI is an index of relative weight rather than body fat and it cannot differentiate the levels of fatness and leanness among individuals. Thirdly, given that sex maturation play important role on the physical fitness performance, we only collected the information on the age of menarche for girls and first nocturnal emission for boys but pubertal stage. However, the age of the study population ranged from 7 to 11 years and 97.4% girls and 99.92% boys were without menarche/nocturnal emission. The exclusion of these participants had no effect on the relationship between weight status and physical fitness (data not shown). In addition, our study is a cross-sectional study which cannot make the conclusion whether obesity causes low fitness or vice versa.

## 5. Conclusions

It is concluded that the overweight and obese children performed worse in cardiorespiratory fitness, muscle explosive strength, and speed compared with normal weight children.

## Figures and Tables

**Table 1:** The prevalence of overweight and obesity in Chinese children aged 6–11 years by gender and age (%).

			Boys				Girls	
Age (years)	*N*	Underweight	Overweight	Obesity	*N*	Underweight	Overweight	Obesity
6	157	2.0	18.5	9.5	178	2.8	12.4	3.4
7	617	1.5	11.0	9.7	598	2.9	9.9	5.3
8	879	0.7	16.0	10.8	771	3.3	14.8	5.3
9	814	2.6	16.7	9.8	771	6.4	16.3	4.9
10	695	1.5	19.4	11.9	638	5.7	13.9	5.8
11	442	4.8	16.7	8.4	369	6.5	11.1	3.8

Total	3604	2.0	16.2	10.3	3325	4.7	13.6	5.1

Significant difference in the prevalence of underweight, overweight, and obesity using Chi-square test between boys and girls in all ages with *P* < .001.

**Table 2:** Physical fitness test results by age and gender (Mean ± SD).

Age	Standing broad jump(cm)		50 m sprint (s)		50 m*8 shuttle run (s)	
(years)	Boys	Girls	Boys	Girls	Boys	Girls
6	122.1 ± 15.7	112.6 ± 16.1	11.2 ± 1.1	11.8 ± 1.2	137.1 ± 15.2	138.9 ± 18.1
7	130.0 ± 17.8	122.4 ± 16.9	10.9 ± 1.2	11.2 ± 1.3	134.2 ± 18.9	135.3 ± 17.6
8	140.1 ± 18.3	130.7 ± 17.7	10.4 ± 1.2	10.8 ± 1.2	129.7 ± 18.4	132.3 ± 16.0
9	149.7 ± 19.8	140.6 ± 18.3	10.1 ± 1.3	10.4 ± 1.1	124.7 ± 18.4	128.7 ± 16.8
10	157.3 ± 20.6	149.2 ± 17.5	9.7 ± 1.3	10.1 ± 1.2	120.8 ± 16.6	123.6 ± 15.6
11	164.5 ± 21.9	156.0 ± 19.3	9.9 ± 1.6	10.1 ± 1.5	119.8 ± 19.0	120.8 ± 13.9

Total	146.1 ± 22.9	136.9 ± 21.6	10.3 ± 1.4	10.6 ± 1.3	127.2 ± 18.8	130.0 ± 17.2

Significant difference in all three physical fitness test results between boys and girls using *t*-test with *P* < .001.

Significant difference in all three physical fitness test results among age groups using ANOVA test with *P* < .001.

**Table 3:** Mean in physical fitness test results for underweight, normal weight, overweight, and obesity group by gender (Mean ± SD).

	Standing broad jump (cm)	50 m sprint (s)	50 m*8 shuttle run (s)
Boys			
Underweight	152.6 ± 23.5	10.2 ± 1.5	123.1 ± 16.5
Normal weight	149.0 ± 22.2	10.1 ± 1.3	123.5 ± 16.7
Overweight	140.6 ± 22.1	10.5 ± 1.4	131.8 ± 20.2
Obesity	132.0 ± 21.1	11.2 ± 1.4	142.1 ± 19.8

Girls			
Underweight	140.0 ± 19.4	10.6 ± 1.1	124.7 ± 15.3
Normal weight	138.1 ± 21.5	10.5 ± 1.3	128.4 ± 16.4
Overweight	132.2 ± 21.2	10.8 ± 1.3	134.6 ± 17.7
Obesity	128.8 ± 21.4	11.2 ± 1.5	141.5 ± 20.2

Significant difference in physical fitness test results among children with normal weight, overweight, and obesity using ANCOVA test with *P* < .001.

**Table 4:** Proportion of children with test result above the age- and gender-specific 75th percentile (P75) and 90th percentile (P90) by body weight status.

	>P75			>P90		
	%	OR	95% CI	%	OR	95% CI
Standing broad jump						
Underweight	32.9	1.22	0.91–1.63	10.2	0.81	0.52–1.27
Normal weight	28.8	1.00	—	12.5	1.00	—
Overweight	14.2	0.41	0.34–0.49	4.6	0.34	0.25–0.46
Obesity	8.5	0.23	0.17–0.32	3.3	0.24	0.15–0.39

50 m sprint						
Underweight	27.4	0.85	0.62–1.15	11.4	0.88	0.57–1.35
Normal weight	30.8	1.00	—	12.7	1.00	—
Overweight	19.4	0.54	0.46–0.64	6.1	0.45	0.34–0.59
Obesity	8.9	0.22	0.16–0.30	2.1	0.15	0.08–0.28

50 m*8 shuttle run						
Underweight	36.1	1.36	0.96–1.91	15.0	1.21	0.77–1.91
Normal weight	29.3	1.00	—	12.9	1.00	—
Overweight	13.9	0.39	0.31–0.48	5.6	0.40	0.29–0.54
Obesity	7.1	0.31	0.23–0.42	2.0	0.13	0.07–0.27
